# The complete chloroplast genome of the traditional Chinese herb, *Uncaria rhynchophylla* (Rubiaceae)

**DOI:** 10.1080/23802359.2019.1703597

**Published:** 2020-01-09

**Authors:** Li-Zhen Ling, Shu-Dong Zhang

**Affiliations:** School of Biological Sciences and Technology, Liupanshui Normal University, Liupanshui, China

**Keywords:** Chloroplast genome, phylogenetic analysis, Rubiaceae, *Uncaria rhynchophylla*

## Abstract

*Uncaria rhynchophylla* is a vine plant belonging to the family Rubiaceae and has been used as medicine for a long time in China. In this study, the complete chloroplast (cp) genome sequence of *U. rhynchophylla* was first reported and characterized. The cp genome was 154,605 bp in length and contains a pair of inverted repeats (IRs, 34,165 bp each) separated by a large (84,327 bp) and small (12,966 bp) single-copy regions. A total of 113 unique genes were predicted, including 79 protein-coding genes, 30 tRNA genes and 4 rRNA genes. The phylogenetic analysis suggested that *U. rhynchophylla* was closer to *Neolamarckia cadamba*.

*Uncaria rhynchophylla* (Miq.) Jacks is a vine plant of the Rubiaceae family. The dry branches bearing hooks of this species known as Gouteng are officially listed in the Chinese Pharmacopeia and are used as an antiaggregation, antidepressant, antipyretic, and anticonvulsant for the treatment of headache, vertigo, and epilepsy (Fujiwara et al. 2006; Geng et al. 2019; Yang et al. 2019). The major active components of *U. rhynchophylla* are alkaloids, terpenoids, and flavonoids (Yang et al. 2019). To provide genomic resources for investigating the evolution of *U. rhynchophylla*, the complete chloroplast (cp) genome of this species was analyzed from high-throughput Illumina sequencing reads.

The fresh leaves of *U. rhynchophylla* were collected from Guiyang (Guizhou, China, N26°37′53″, E106°43′23″, 1,280 m) and the specimen (lpssy0287) was deposited in the herbarium of the Liupanshui Normal University (LPSNU). The genomic DNA was extracted and sequenced as previously described (Zhang et al. 2019). Approximately 2 Gb raw data were generated and used for *de novo* cp genome assembly with SPAdes (Bankevich et al. 2012) and all predicted genes were annotated using PGA (Qu et al. 2019). The complete cp genome sequence of *U. rhynchophylla* was deposited in GenBank database (accession number MN723865).

The complete *U. rhynchophylla* cp genome is 154,605 bp in length, including a large single copy (LSC) region of 84,327 bp, a small single copy (SSC) region of 12,966 bp, and a pair of inverted repeats (IRs) of 34,165 bp each. The cp genome shows the GC content of 37.7% and contains 113 unique genes, including 79 protein-coding genes, 30 transfer RNA (tRNA) genes, and four ribosomal RNA (rRNA) genes. Most of these genes are in a single copy; however, four rRNA genes (4.5S, 5S, 16S and 23S rRNA), six protein-coding genes (*ndhB*, *rpl2*, *rpl23*, *rps12*, *rps7*, and *ycf2*), and seven tRNA genes (*trnA-UGC*, *trnI-CAU*, *trnI-GAU*, *trnL-CAA*, *trnN-GUU*, *trnR-ACG*, and *trnV-GAC*) occur in double copies. Fifteen distinct genes (*atpF*, *ndhA*, *ndhB*, *petB*, *petD*, *rpl16*, *rpl2*, *rpoC1*, *rps16*,* trnA-UGC*, *trnG-GCC*, *trnI-GAU*, *trnK-UUU*, *trnL-UAA*, and *trnV-UAC*) contain one intron and three genes (*clpP*, *rps12*, and *ycf3*) have two introns.

Rubiaceae is one of the five largest families of flowering plants with over 13,000 species and is divided into three subfamilies (subfam. Cinchonoideae, subfam. Ixoroideae and subfam. Rubioideae) and over 63 tribes (Stevens 2001; Bremer 2009). To understand the phylogenetic position of *Uncaria* within the family Rubiaceae, *U. rhynchophylla* and other eight genera (*Galium*, *Paederia*, *Gynochthodes*, *Emmenopterys*, *Coffea*, *Antirhea*, *Mitragyna*, and *Neolamarckia*) representing seven tribes of Rubiaceae were used for phylogenetic analysis based on their complete cp genomes. Seven species from the other families of Gentianales were used for outgroups. The phylogenetic tree was constructed by maximum likelihood (ML) and Bayesian inference (BI) methods using RAxML (Stamatakis 2014) and MrBayes (Ronquist et al. 2012). As can be seen from Figure 1, a framework of the phylogeny with support for three subfamilies was obtained. *Uncaria rhynchophylla* was closer to *Neolamarckia cadamba*, which was formed a sister group with another Naucleeae tribe species, *Mitragyna speciose*.

**Figure 1.**
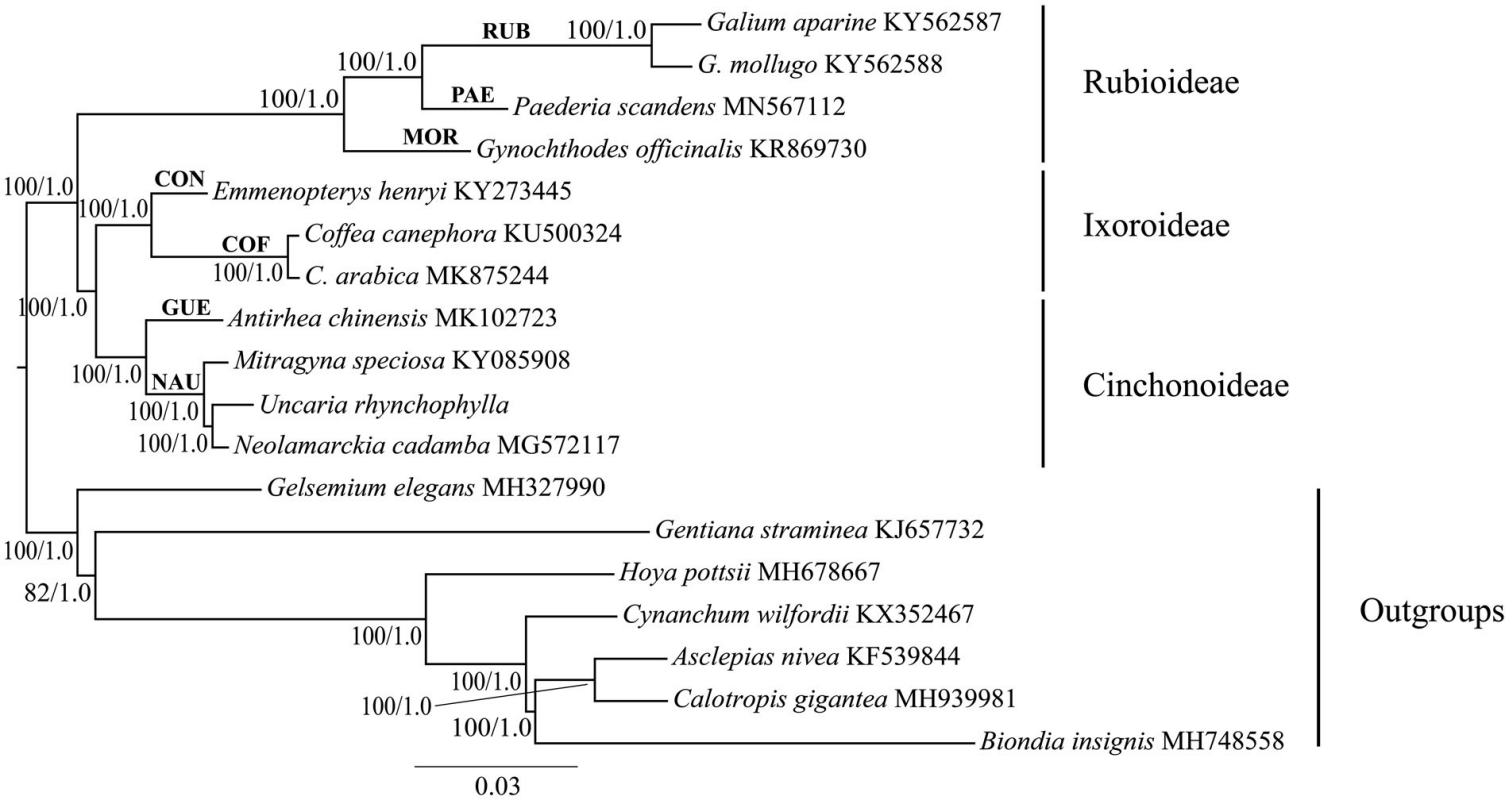
The maximum likelihood (ML) tree of 11 species from the Rubiaceae family inferred from the complete chloroplast genome sequences. Numbers at nodes correspond to ML bootstrap percentages (1,000 replicates) and Bayesian inference (BI) posterior probabilities. COF: Coffeeae; CON: Condamineeae; GUE: Guettardeae; MOR: Morindeae; NAU: Naucleeae; PAE: Paederieae; RUB: Rubieae.
